# *Viburnum opulus* L. Juice Phenolics Inhibit Mouse 3T3-L1 Cells Adipogenesis and Pancreatic Lipase Activity

**DOI:** 10.3390/nu12072003

**Published:** 2020-07-06

**Authors:** Małgorzata Zakłos-Szyda, Nina Pietrzyk, Marcin Szustak, Anna Podsędek

**Affiliations:** Department of Biotechnology and Food Sciences, Institute of Molecular and Industrial Biotechnology, Lodz University of Technology, 90-924 Łódź, Poland; nina.pietrzyk@dokt.p.lodz.pl (N.P.); marcin.szustak@p.lodz.pl (M.S.); anna.podsedek@p.lodz.pl (A.P.)

**Keywords:** *Viburnum opulus*, phenolic compounds, adipogenesis, PPARγ, lipase inhibition

## Abstract

*Viburnum opulus* L. fruit is a rich source of phenolic compounds that may be involved in the prevention of metabolic diseases. The purpose of this study was to determine the effects of *Viburnum opulus* fresh juice (FJ) and juice purified by solid-phase extraction (PJ) on the adipogenesis process with murine 3T3-L1 preadipocyte cell line and pancreatic lipase activity in triolein emulsion, as well as their phenolic profiles by UPLC/Q-TOF-MS. Decrease of lipids and triacylglycerol accumulation in differentiated 3T3-L1 cells were in concordance with downregulation of the expression of peroxisome proliferator-activated receptor-gamma (PPARγ), CCAAT/enhancer-binding protein alpha (C/EBPβ/α), and sterol regulatory element-binding protein 1c (SREBP-1c). Furthermore, regulation of PPARγ-mediated β-lactamase expression by *V. opulus* components in reporter gene assay, as well as their binding affinity to ligand-binding domain of PPARγ, were tested. In addition, the levels of enzymes involved in lipid metabolism, like fatty acid synthase (FAS) or acetyl-CoA carboxylase (ACC), were decreased, along with inflammatory cytokines, like tumor necrosis factorα (TNFα), interleukin-6 (Il-6) and leptin. Moreover, FJ and PJ were able to inhibit pancreatic lipase, which potentially could reduce the fat absorption from the intestinal lumen and the storage of body fat in the adipose tissues. Thirty-two phenolic compounds with chlorogenic acid as the dominant compound were identified in PJ which revealed significant biological activity. These data contribute to elucidate *V. opulus* juice phenolic compounds’ molecular mechanism in adipogenesis regulation in 3T3-L1 cells and dietary fat lipolysis.

## 1. Introduction

Nutrition-related chronic diseases have become the main health problem of the world of the 21st century. According to World Health Organization data based on the latest, in the European Union countries overweight and obesity affect 30–70% and 10–30% of adults, respectively [1]. Obesity is characterized as abnormal or excessive fat accumulation, which means the increase in the number (hyperplasia) and size (hypertrophy) of differentiated adipocytes [2,3]. It is known that adipose tissue is not only a reservoir of energy in the form of triacyclglycerols (TAG), but it also secretes adipocytokines, growth factors, and hormones involved in energy homeostasis and insulin sensitivity maintenance. Thus, increased adiposity is regarded as one of the most important risk factors of insulin resistance, type 2 diabetes (T2D), and nonalcoholic fatty liver disease (NAFLD), which in turn may lead to hypertension, coronary heart disease, and stroke [4]. Among the most important cellular regulators of lipid metabolism are peroxisome proliferator-activated receptors (PPARs), belonging to nuclear receptors proteins [5]. There are three different receptor isotypes: PPARα, PPARβ/δ, and PPARγ, among which PPARγ is involved in the regulation of adipocyte differentiation. Ligand binding to the PPARγ ligand-binding domain (LBD) leads to a conformational change and switching of nuclear receptor corepressors to coactivators [6]. Agonists activate PPARγ, which, after binding with retinoic X receptor and peroxisome proliferator responsive element (PPRE) within the promoter of target genes activates their transcription [7]. Not only PPARγ but also other transcription factors, such as CCAAT/enhancer-binding protein alpha (C/EBPβ/α) and sterol regulatory element binding protein-1c (SREBP-1c), are involved in adipocyte differentiation [8]. These factors stimulate gene expression and the expression of other proteins involved in lipid synthesis and storage, such as fatty acid synthase (FAS) or acetyl-CoA carboxylase (ACC). What is more, obese adipose tissue contributes to the elevation of inflammatory cytokines, such as tumor necrosis factor α (TNFα) or interleukin-6 (Il-6), leading to chronic inflammation state promoting insulin resistance and cancer development [9]. PPARγ agonist type of medicines, like glitazones, significantly improve glycemic control, but via promotion of free fatty acid uptake and accumulation of TAG in adipose tissue lead to weight gain, and increases in heart or renal failure [10,11,12]. Thus, due to the variety of induced side effects, less harmful plant-derived agents are searched as PPARγ regulators able to prevent insulin resistance without weight obesity gaining. Other mechanisms allowing the decrease of fatty acids intestinal absorption is the usage of pancreatic lipase inhibitor, which decreases the hydrolysis of diet-originated TAG into glycerol and fatty acids. Since phenolic compounds constitute an important part in the human diet, they have recently emerged as critical phytochemicals in obesity prevention and treatment [13,14]. Among fruits rich in these secondary metabolites are those of *Viburnum opulus* L. (*V. opulus* L.), known as guelder rose, or the European cranberry bush rose [15,16]. Despite its fruit bitterness it can be found in food products such as juice, jams, jellies, marmalades, sauces, herbal tea, cordials, and liqueurs, as well as fermented drinks [17]. Our previous studies showed that due to their high antioxidant potential *V. opulus* phenolics decreased chemically generated intracellular oxidative stress under *in vitro* conditions, as well as possessed different anticancer activity with apoptosis induction and inhibition of cell migration [16,18,19]. Further studies revealed *V. opulus* fruit phenolics’ impact on carbohydrate metabolism as α-amylase, α-glucosidase, protein tyrosine phosphatase-1B, and dipeptidyl peptidase-4 enzyme inhibitors [20,21]. More detailed analysis showed that *V. opulus* phenolics decreased the uptake of free fatty acids and lipids accumulation in human epithelial Caco-2 cells [15]. Nevertheless, they inhibited glucose-stimulated insulin secretion in mice insulinoma MIN6 cells, as well as increased free fatty acid uptake and lipid droplets accumulation [21]. Taking into account potent phenolics’ impact on the modulation of cellular metabolism, in the present study we investigated an influence of *V. opulus* fresh juice (FJ) and purified juice (PJ) on pancreatic lipase activity and adipogenesis process in mouse preadipocyte 3T3-L1 cell line [22]. In addition, phenolic components of FJ and PJ were identified by the UPLC-MS method. Studies also assessed the influence of these preparations on the expression of transcription factors (PPARγ, C/EBP, SREBP1c) and other proteins related to adipogenesis (FAS, ACC, TNFα, Il-6, leptin, adiponectin). Furthermore, the *V. opulus* components’ influence on the regulation of PPARγ activity was elucidated.

## 2. Materials and Methods

### 2.1. Chemicals and Reagents

Acetonitrile (Merck, Darmstadt, Germany) and formic acid (Sigma-Aldrich, Steinheim, Germany) were hyper grades for LC-MS. Folin–Ciocalteu reagent was obtained from POCH (Gliwice, Poland). The reference compounds were obtained from Sigma-Aldrich (Steinheim, Germany) ((+)-catechin, (−)-epicatechin, rutin, gallic acid), Extrasynthese (Lyon, France) (chlorogenic acid, cyanidin 3-glucoside, quercetin 3-glucoside, isorhamnetin, and isorhamnetin 3-rutinoside) and Phytolab (Vestenbergsgreuth, Germany) (neochlorogenic acid, procyanidin B1, and procyanidin B2). Ultrapure water (Simplicity® Water Purification System, Millipore, Marlborough, MA, USA) was used to prepare all the solutions. All cell culture reagents were obtained from Life Technologies (Carlsbad, CA, USA). Other chemicals used, if not stated otherwise, were obtained from Sigma-Aldrich (Steinheim, Germany).

### 2.2. Preparation of V. opulus Samples, Identification and Quantitative Determination of Individual Phenolic Compounds by UPLC–PDA-Q/TOF-MS

Fruits of the *V. opulus* were collected from Rogów Arboretum, Warsaw University of Life Sciences (Rogów, Poland) (account number 18162). After fruit pulp homogenization and centrifugation (5000 rpm for 10 min), fresh juice (FJ) was obtained. FJ purification by solid phase extraction with C-18 Sep-Pak cartridge (10 g capacity, Waters Corp., Milford, MA, USA; 12-Port Vacuum Manifold system) and methanolic elution processes were performed. After methanol removal under reduced pressure (T < 40 °C), solid residue was dissolved in water and lyophilized to purified juice (PJ). Phenolic compounds were identified using the Acquity ultraperformance liquid chromatography (UPLC) system coupled with a quadruple-time of flight mass spectrometry (Q/TOF-MS) instrument (Waters Corp., Milford, MA, USA) equipped with an electrospray ionization (ESI) source. The separation of individual phenolics was carried out using an Acquity UPLCR HSS T3 C18 column (150 × 2.1 mm, 1.8 µm; Corp., Milford, MA, USA) at 30 °C. The mobile phase was a mixture of 0.1% formic acid (A) and acetonitrile (B). The gradient program was as follows: initial conditions 99% (A), 12 min 65% (A), 12.5 min 100% (B), 13.5 min 99% (A). The flow rate was 0.45 mL/min and the injection volume was 5 µl. The mass spectrometer was operating in the negative mode for a mass range of 150–1500 Da, fixed source temperature at 100 °C, desolvation temperature 250 °C, desolvation gas flow of 600 L/h, cone voltage of 45 V, a capillary voltage of 2.0 kV, and a collision energy of 50 V. Leucine enkephalin was used as a lock mass. The instrument was controlled by Mass-LynxTM V 4.1 software (Waters Corp., Milford, MA, USA). The runs were monitored at the following wavelengths: flavanols at 280 nm, hydroxycinnamic acids at 320 nm, flavonols at 360 nm, and anthocyanins at 520 nm. Photodiode detector (PDA) spectra were measured over the wavelength range of 200–600 nm. Calibration curves were run for the external standards: (+)-catechin, procyanidin C1, neochlorogenic acid, chlorogenic acid, cryptochlorogenic acid, caffeic acid, and quercetin 3-rutinoside, and quercetin 3-glucoside. Phenolic compounds were identified using their UV-Vis characteristic, MS and MS2 properties using data gathered in-house and from the literature.

### 2.3. Inhibition Assay for Pancreatic Lipase Activity

Inhibitory activities of FJ and PJ were expressed as the IC_50_ values (half-maximal inhibitory concentration). Orlistat was used as a positive inhibitor control. The IC_50_ value was concluded from the graph of lipase inhibition (%) versus the concentration of juices or Orlistat per 1 mL of the reaction mixture under assay conditions. The pancreatic lipase activity was tested by measuring the fatty acids released from emulsified triolein (0.6 g of triolein, 25 mL of Tris-buffer, and 0.4 g of bile acids) according to the method described previously [23]. Briefly, 0.3 mL of FJ, PJ, and orlistat solutions diluted with buffer were mixed with 0.5 mL of the triolein emulsion and pre-incubated at 37 °C for 5 min before adding lipase supernatant (0.063 mL). Blanks with buffer instead of the lipase supernatant were prepared for background correction. The control consisted of all solutions without inhibitor. Finally, the reaction mixtures were incubated in a shaking bath (200 rpm) at 37 °C for 30 min. The reaction was terminated by adding 0.23 mL of HCl. Then, 3 mL of isooctane was added and vortexed for 0.5 min. The upper layer (2 mL) was collected, followed by the addition of 0.4 mL copper reagent (5% copper acetate, pH 6.1 regulated by pyridine). After vortexing for 1 min, the upper layer was centrifuged at 10,000 rpm for 10 min and its absorbance (A) was measured at 720 nm against a reagent blank. All samples were assayed in triplicate. Percent inhibition of pancreatic lipase activity was calculated using the formula:

Lipase inhibition (%) = [(A_c_ − A_cb_) − (A_s_ − A_sb_)] / (A_c_ − A_cb_) × 100 (1)

where A_c_ is the absorbance of the control, A_cb_ is the blank control absorbance, A_s_ is the sample absorbance, A_sb_ is the sample blank.

### 2.4. Cell Culture and Exposure Conditions

Mouse preadipocytes 3T3-L1 were supplied by ATCC (Manassas, VA, USA). Preadipocytes were grown in Dulbecco′s Modified Eagle′s Medium (DMEM) medium with high glucose supplemented with 10% bovine calf serum. For adipocyte differentiation a confluent culture of 3T3-L1 cells was grown for two days in a preadipocyte medium DMEM with 10% calf serum, then the cells were stimulated with a differentiation medium with DMEM containing 10% fetal bovine serum (FBS), 1 µM dexamethasone, 0.5 mM methylisobutylxanthine (IBMX), and 1 µg/mL insulin for two days. After 48 h of incubation, the differentiation medium was replaced with DMEM containing 10% FBS and 1 µg/mL insulin [22]. Cell medium was replaced at 2-day intervals with the addition of compounds studied. Analyses were carried out 7 days after differentiation if not stated otherwise. To perform biological activity assays, a stock solution of PJ at concentration 100 mg/mL in 50% dimethyl sulfoxide (DMSO) was prepared and further dilutions were made with culture medium. The sample’s concentrations used in biological studies are presented in the descriptions of the tests carried out. All cell culture experiments were performed in a humidified 5% CO_2_ and 95% atmosphere at 37 °C. Tissue culture plastics were supplied by Greiner Bio-One GmbH (Frickenhausen, Austria). All the experimental measurements were performed using the Synergy 2 BioTek Microplate Reader (BioTek, Winooski, VT, USA). Microscopic observations were performed using contrast-phase and fluorescent microscope Nikon TS100 Eclipse (Nikon, Tokyo, Japan) under 200 × magnification, if not stated otherwise.

### 2.5. Cell Viability

The effects of FJ and PJ on cell viability were assayed with the PrestoBlue reagent. The 3T3-L1 preadipocytes were seeded into a 96-well plate at a density of 10^4^ cells/well overnight. Two days after confluence, cells were treated with series of extracts concentrations for 48 hours. The final concentration of DMSO did not exceed 0.005%. Then, the PrestoBlue reagent was added for 30 min and fluorescent signal at F530/590 nm was measured. For cell visualization, 2 µM calcein AM (Thermo Fisher Scientific, Waltham, MA, USA) was directly added to the cells.

### 2.6. Detection of Intracellular Reactive Oxygen Species Generation

The 3T3-L1 preadipocytes were seeded into a 96-well plate at a density of 10^4^ cells/well. After the cells’ treatment with extracts, the cells were washed with phosphate buffer saline (PBS) and incubated with DMEM and 10 μM of dichloro-dihydro-fluorescein diacetate (DCFH-DA) dye. Fluorescence intensity at F485/530 nm was determined after 30 min incubation. Five-hundred μM *tert*-BOOH (*t*-BOOH) was used as a positive control. The intracellular fluorescence of cells was observed after cells treatment with chemicals under a fluorescence microscope.

### 2.7. Determination of Lipid Accumulation, Free Fatty Acid Uptake and Triglyceride Content

The 3T3-L1 preadipocytes were seeded into a 96-well plate at a density of 10^4^ cells/well for each of the experiment. The lipid content in the mature adipocytes was determined using the Nile red staining method. After cell incubation with FJ and PJ, cells were washed with cold PBS and fixed in 5% paraformaldehyde for 30 min. Then, the cells were stained with Nile red (1 μg/mL) for 40 min and fluorescence intensity at F485/530 nm was measured. For cell nuclei visualization, to fixed cells, 1 μg/mL 4′,6-Diamidine-2′-phenylindole dihydrochloride (DAPI) stain was added. The measurement of fatty acid fluorescent probe TF2-C12 uptake by cells was performed with the Fatty Acid Uptake Kit (Sigma-Aldrich, Seinheim, Germany). After the cells’ treatment with the preparations, the fluorescent signal at F485/530 nm was measured after 1 h incubation with fluorescent analogue. 

Triglyceride content was measured using the Triglyceride Colorimetric Assay kit (Cayman Chemical, Ann Arbor, MI, USA). To perform the experiment cells were seeded into a 6-well plate at a density of 2 × 10^5^ cells/well. Following treatment, the differentiated 3T3-L1 adipocytes were rinsed with PBS, harvested with a cell scraper, lysed with 1% Triton X-100 and the total triglyceride content was assessed according to the manufacturer’s instructions with an absorbance measurement at 540 nm.

### 2.8. Measurement of Adipolysis

To perform, the experiment cells were seeded into a 24-well plate at a density of 4 × 10^4^ cells/well. After treatment with FJ and PJ, the cells were washed with PBS and incubated with an induction solution for 1 h. Then the medium was collected, and glycerol released into the medium was measured at 570 nm using a colorimetric assay Adipolysis Assay Kit (Sigma-Aldrich, Steinheim, Germany) and following the manufacturer’s instructions. As a positive control (lipolysis inducer), 10 µM isoproterenol was used.

### 2.9. Gene Expression Analysis

To perform, the experiment cells were seeded into a 6-well plate at a density of 2 × 10^5^ cells/well. After cell incubation with FJ and PJ, the total RNA was extracted with the GeneMatrix Universal RNA Purification Kit (Eurex Ltd., Gdansk, Poland) according to the manufacturer’s procedure. RNA samples were purified with an amplification Grade DNase I (Sigma-Aldrich, Steinheim, Germany), and reverse transcribed with the NG dART RT Kit (Eurex Ltd., Gdansk, Poland). Real-time RT-PCR was carried out using the SG qPCR Master Mix (Eurex Ltd., Gdansk, Poland) on a BioRad CFX96 qPCR System (Bio-Rad, Hercules, CA, USA). Complementary DNA representing 6 ng of total RNA per sample was subjected to 40 cycles of PCR amplification. Samples were first incubated at 95 °C for 40 s, then at 55 °C for 30 s, and finally at 72 °C for 30 s. To exclude non-specific products and primer-dimers, after the cycling protocol, a melting curve analysis was performed by maintaining the temperature at 52 °C for 2 s, followed by a gradual temperature increase to 95 °C. The threshold cycle (Ct) values for that gene did not change in independently performed experiments. The level of target gene expression was calculated as 2^−ΔΔCt^, where ΔΔCt = [Ct(target) − Ct(*β*actin)]sample − [Ct(target) − Ct(*β*actin)]. The following primer sequences were used to determine the genes’ expression: CREB-binding protein (*CBP*): forward primer (F) TTACAACAGGCCAGGTTTCC, reverse primer (R) GGCTGGCGACATACAGTACA; sterol regulatory element binding transcription factor 1 (*SREBP1*): (F) TGTTGGCATCCTGCTATCTG, (R) AGGGAAAGCTTTGGGGTCTA; *β-actin*: (F) CCACAGCTGAGAGGGAAATC, (R) AAGGAAGGCTGGAAAAGAGC; *adiponectin*: (F) AGATGGCACTCCTGGAGAGAAG, (R) ACATAAGCGGCTTCTCCAGGCT; *leptin*: (F) GGATCAGGTTTTGTGGTGCT, (R) TTGTGGCCCATAAAGTCCTC; fatty acid synthase (*FAS*): (F) TTGCTGGCACTACAGAATGC, (R) AACAGCCTCAGAGCGACAAT; peroxisome proliferator activated receptor gamma (*PPARγ*): (F) GCGGAAGAAGAGACCTGGG, (R) AGAACGTGACTTCTCAGCCC; interleukin-6 (*Il-6*): (F) GTCCTTCCTACCCCAATTTCCA, (R) TAACGCACTAGGTTTGCCGA; CCAAT/enhancer binding protein (*C/EBP*) (F) GTGTGCACGTCTATGCTAAACCA, (R) GCCGTTAGTGAAGAGTCTCAGTTTG; tumor necrosis factor α (*TNFα*): (F) GGGATCTGCTCCGCGGTTGT, (R) TCCGCGGCCAGGAGAACTGT; acetyl-Coenzyme A carboxylase alpha (*ACC*): (F) GGGGATCTCTGGCTTACAGG, (R) ATCGCATGCATTTCACTGCT; fatty acid translocase (*FAT/CD36*), (F) TGGCCTTACTTGGGATTGG, (R) CCAGTGTATATGTAGGCTCATCCA.

### 2.10. Western Blotting

To perform the experiment cells were seeded into a 6-well plate at a density of 2 × 10^5^ cells/well. To prepare the total cell lysates, monolayers of 3T3-L1 adipocytes were scraped and lysed in Mammalian Protein Extraction Reagent (M-PER) containing protease and phosphatase inhibitors cocktail (Thermo Scientific, Waltham, MA, USA). Then, the lysates were centrifuged at 13,000 rpm for 5 min, and the supernatants of cell lysates were separated. The protein quantification was measured using the Protein Assay Dye Reagent Concentrate (Bio-Rad Laboratories BmbH, München, Germany). Each 20 µg of protein samples were separated by 8% or 10% sodium dodecyl sulphate-polyacrylamide gel electrophoresis (SDS-PAGE) and transferred to 0.45 µm nitrocellulose blotting membrane (GE Healthcare, Chicago, IL, USA). The membranes were blocked using 5% bovine serum albumin (BSA) in tris-buffered saline containing 0.1% Tween-20 (TBST) for 2 h at room temperature and incubated with primary antibodies overnight at 4 °C diluted 1:1000 in the same solution. Polyclonal rabbit antibodies targeting PPARγ (#2435), RxRα (#5388), CBP (#7389), acetyl-CoA carboxylase (#3676), phospho-acetyl-CoA carboxylase (Ser79) (#3661), phospho-AMPKα (Thr172) (#2531) and *β*actin (#4967) were purchased from Cell Signaling Technology (Danvers, MA, USA), SREBP-1c (14088-1-AP) from Proteintech Group (Manchester, UK), and p-IRS-1 (Ser307) from Santa Cruz Biotechnology (Dallas, TX, USA). Afterwards, the membranes were washed three times with TBST, then incubated for 1 h at room temperature with horseradish peroxidase (HRP)-conjugated secondary anti-rabbit antibody (#7074, Cell Signaling Technology, Danvers, MA, USA) diluted 1:3000 in 5% nonfat dry milk in TBST. After that membranes were rewashed three times with TBST. The proteins were visualized using an enhanced chemiluminescent SuperSignal West Pico Trial Kit (Thermo Scientific, Waltham, MA, USA). The ChemiDocTM MP Image System with Image LabTM 5.1 software (Bio-Rad Laboratories, Hercules, CA, USA) was used for acquisition and densitometric analysis of western blot images. Relative protein band intensity was normalized to *β*-actin and quantified with respect to control cells.

### 2.11. Determination of Selected Proteins Levels

To perform, the experiment cells were seeded into a 6-well plate at a density of 2 × 10^5^ cells/well. On the last day of cells treatment the medium was collected and protein concentrations of adiponectin (Adiponectin Mouse ELISA Kit, Abcam, Cambridge, GB), leptin (Leptin Mouse ELISA Kit, Abcam, Cambridge, GB), Il6 (Mouse IL6 ELISA kit, Biorbyt Ltd., Cambridge, GB) and TNFα (Mouse TNF alpha ELISA kit, Biorbyt Ltd., Cambridge, GB), were determined using ELISA kits, following the manufacturer’s instructions. The PPARγ protein level present in the nuclear fraction of the cellular lysates was determined with the PPARγ Transcription Factor Assay Kit (Abcam, Cambridge, GB).

### 2.12. Reporter Gene Assay

Cellular activation of the PPARγ nuclear receptor was assessed in the reporter gene assay GeneBLAzer® PPAR gamma 293H DA (Invitrogen, Carlsbad, CA, USA) according to the manufacturer’s protocol. In brief, cells stably expressing specific PPARγ ligand-binding domain fusion protein and UAS-β-lactamase reporter gene were incubated with tested extracts for 16 h, the cells were loaded with cell-permeable LiveBLAzer FRET B/G substrate, after 2 h incubation fluorescence intensities at 460 and 530 nm emission following excitation at 420 nm were measured. After subtraction of fluorescence background from cell-free wells, the ratio of fluorescence intensity at 460 versus 530 nm was calculated. As the PPARγ inhibitor 0.1 µM T0070907 was used, whereas 1 µM rosiglitazone as the activator.

### 2.13. Molecular Modeling

To determine the potential interaction between analyzed FJ or PJ components and the PPARγ receptor, molecular docking simulation was performed. An X-ray crystal structure for human PPARγ receptor in complex with rosiglitazone (5YCP) was obtained from the Protein Data Bank database (http://www.rcsb.org/) as a .pdb file [24]. Subsequently, the protein molecule model was prepared to docking procedure (hydrogen addition and grid box coordinates determination) with the AutoDock Tolls software. Then the protonation state of the side chains was defined with the PROPKA software (available at http://nbcr-222.ucsd.edu/pdb2pqr_2.0.0/). Structures of rosiglitazone, chlorogenic acid, and (+)- catechin were downloaded from the ZINC database (http://zinc.docking.org/substances/home/). The procyanidin B1 and C1 structures were built with ChemSkech software. The obtained files were converted to .pdb file with the OPENBABEL tools http://www.cheminfo.org/Chemistry/Cheminformatics/FormatConverter/index.html). Then, all the ligand molecules were prepared for further modeling (determine rotating bonds) with the AutoDock Tools and saved as .pdbqt file. The docking of the prepared ligand to PPARγ receptor was performed with Autodock Vina docking software. [AutoDock Vina: improving the speed and accuracy of docking with a new scoring function, efficient optimization and multithreading]. The size of the grid box was set to 34 × 30 × 30 Å from the center of the binding pocket. The value of the exhaustiveness parameter was set to 150. Images of the docked ligands were shown with the use of the AutoDock Tools software.

### 2.14. Statistical Analysis

Unless stated otherwise, all the biological results are presented as means of 3–6 repeated experiments ± SEM. All calculations were evaluated for significance using one-way ANOVA followed by Dunnett’s test with the GraphPad Prism 6.0 software (GraphPad Software, Inc., La Jolla, CA, USA). *p* ≤ 0.05 was considered statistically significant.

## 3. Results and Discussion

### 3.1. Identification and Content of Phenolic Compounds in V. opulus FJ and PJ with the UPLC–PDA-Q/TOF-MS Method

**Figure 1:**
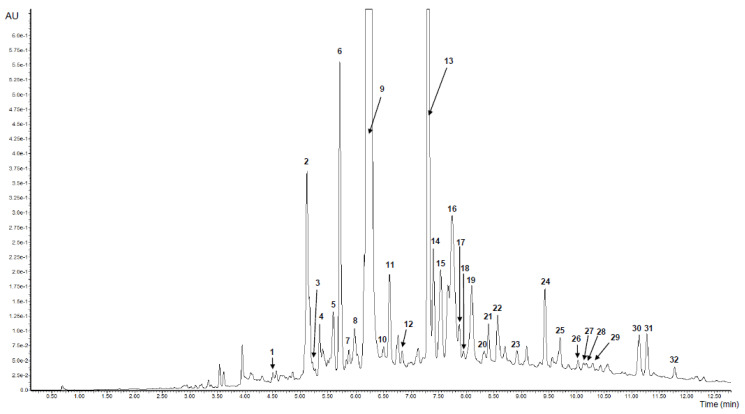
UPLC chromatogram of purified *V. opulus* juice (PJ) at 280 nm. Refer to Table 1 for the identification of each numbered peak.

The results of the qualitative and quantitative analysis of *V. opulus* FJ and PJ determined by the UPLC/MS method are presented in Figure 1 and Table 1 and Table 2. A total of 29 and 30 phenolic compounds were positively identified in FJ and PJ, respectively, on the basis of the complementary information provided by PDA and the ESI-MS detection, and literature data. The FJ and PJ showed the presence of different groups of phenolic compounds, such as hydroxycinnamic acids, flavanols, flavonols, and anthocyanins. Significant differences (*p* ≤ 0.05) in the content of individual phenolic compounds between FJ and PJ were noted as a result of about a 90-fold increase in the content of phenolics in PJ (878.6342 mg/g) compared to FJ (11.508 mg/g). Thus, it could be concluded that the Solid-Phase Extraction (SPE) method on a Sep-Pak C18 column is efficient for removing non-phenolic compounds from the juice. In the published studies, total phenolic determined by the Folin-Ciocalteu method content in the *V. opulus* fruit juices varied from 5.47 to 11.70 mg/g [25].

Hydroxycinnamic acids were the most predominant phenolic group found in both samples and constituted 80.46% and 77.50% of the sum of the phenolics in PJ and FJ, respectively. Chlorogenic acid (peak 9) with the negative molecular ion [M − H]- of 353 with typical fragments of quinic acid ester (m/z at 191) showed the highest content, both among phenolic compounds and hydroxycinnamic acids. Its concentrations were 645.492 mg/g and 8.039 mg/g in PJ and FJ, respectively. Chlorogenic acid, as the main phenolic compound in fresh berries of *V. opulus* and also in fresh juice, have been reported previously, where it constituted more than 96.2% of hydroxycinnamic acid derivatives [1,2,3,4]. Quantitatively, a second compound from hydroxycinnamic acids identified in FJ and PJ was caffeoylquinic acid (peak 13) with the negative molecular ion [M – H]- of 353 and fragment ions m/z at 191, 133. Additionally, two caffeoylquinic acids with the known structures, such as neochlorogenic acid (peak 1) and cryptochlorogenic acid (peak 10), were identified. Besides, five caffeoylquinic acid derivatives (peaks 3, 4, 7, 21, 22) were present in both samples. On the other hand, feruloylquinic acids (peaks 24 and 27) were only identified in PJ. Quantitatively, the second phenolic subgroup of phenolics was flavanols, which constituted 19.52 and 16.30% of FJ and PJ total phenolics, respectively (Table 2). The main flavan-3-ol isomers were (+)-the catechin (peak 6) and procyanidin dimer B1 (peak 2). Also, the procyanidin dimer B2 (peak 11) and procyanidin trimer C1 (peak 17) were identified in both samples based on reference substances. Furthermore, other procyanidin dimer and trimer, which were identified previously, were presented in our samples [26].

*V. opulus* fruits are characterized by varied content of anthocyanins, with cyanidin glycosides, such as glucoside, rutinoside and sambubioside as the main pigments [15,27]. In the tested FJ and PJ, cyanidin-3-glucoside (peak 16) was the main anthocyanin with the content 13.583 mg/g for PJ and 0.139 mg/g of FJ. Content of cyanidin-3-sambubioside (peak 15) and cyanidin-3-rutinoside (peak 19) were similar in both samples. The total content of anthocyanins was 0.300 mg/g of FJ and 25.665 mg/g of PJ. Perova et al. identified ten cyanidin glycoside in *V. opulus* fruits with cyanidin-3-glucoside and cyanidin-3-xylosyl-rutinoside as the main compounds [15]. Among the phenolic compounds estimated, flavonols occurred at the lowest concentration with 2.827 mg/g of PJ and only 0.043 mg/g of FJ (Table 2). *V. opulus* PJ and FJ contained quercetin-3-vicianoside (peak 25), quercetin-3-rutinoside (peak 29), quercetin-3-rhamnoside (peak 32) and quercetin-3-galactoside (peak 28), whereas the latter has not been determined in FJ. There are reports that also isorhamnetin glycosides have been found in *V. opulus* fruit and juice [15,18].

### 3.2. Inhibition of Pancreatic Lipase by V. opulus FJ and PJ

**Figure 2:**
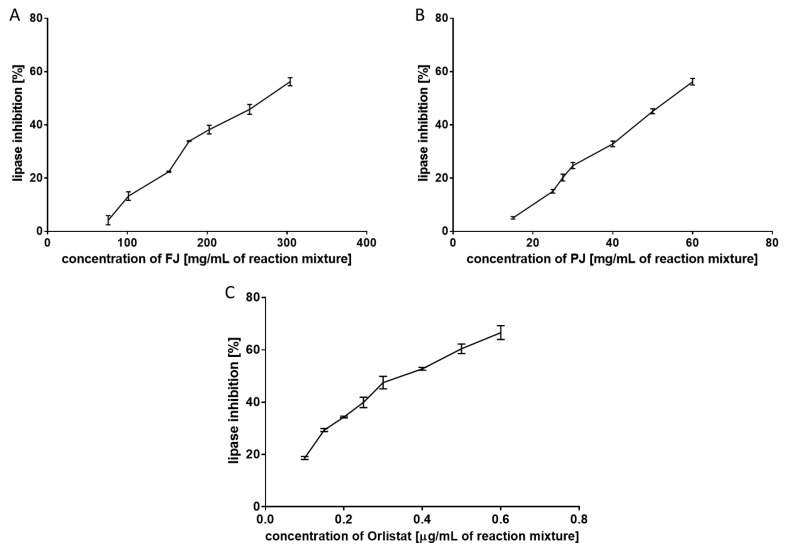
Inhibitory effect of fresh juice (FJ) (**A**), purified juice (PJ) (**B**), and Orlistat (**C**) on pancreatic lipase. Data are means of triplicate assays ± standard deviations.

Pancreatic lipase (EC 3.1.1.3; triacylglycerol acyl hydrolase) breaks down TAG into absorbable monoacylglycerols and free fatty acids. Pancreatic lipase is responsible for the hydrolysis of 50–70% of total dietary fats in the intestinal lumen [28]; thus, its inhibition could reduce the storage of body fat in the adipose tissues. In the present study, the effect of FJ and PJ on pancreatic lipase was determined in a triolein emulsion by the spectrophotometric method with a copper reagent. This lipid substrate is a triglyceride formed by esterification of the three hydroxy groups of glycerol with oleic acid. As shown in Figure 2, both *V. opulus* samples exhibited a dose-dependent inhibitory effect on pancreatic lipase activity. The concentration of PJ inhibiting lipase activity to 50% was equal to 55.26 ± 2.54 mg/mL and showed 4.7-fold higher inhibitory activity than FJ (IC_50_ = 261.94 ± 2.00 mg/mL). All these samples were less potent in pancreatic lipase inhibiting than Orlistat (a well-known anti-lipase agent). The IC_50_ value for lipase inhibition for Orlistat was 0.380 ± 0.004 µg/mL. The lower anti-lipase activity of plant extracts than Orlistat is associated with the presence of other components in the extracts that do not affect the activity of the enzyme. In addition, fruit juice contains several classes of phenolic compounds, which in combination might differently affect the lipase activity due to the different interactions between them, as well as with other fruit components.

In contrast to the widely studied various fruits, literature data on the anti-lipase activity of juices and drinks are scarce. Gironés-Vilaplana et al. demonstrated a significant increase in isotonic citric acid drink and isotonic lemon juice drinkability to inhibit pancreatic lipase after enriching them in açai, blackthorn or maqui berries [29]. Magui berry exhibited the most potent inhibitory activity, and the anti-lipase properties of the drinks were positively correlated with anthocyanins amounts. A similar relationship was found by Fabroni and others [30]. Among 13 fruit extracts, juices, plant tissues, legume seeds, cereals, and vegetables, "Moro" orange juice with the highest anthocyanin content, had the strongest inhibitory effect on pancreatic lipase. The orange juice lipase inhibition IC_50_ value of 0.46 mg/mL was higher than the IC_50_ of Orlistat (0.064 mg/mL). In the present study, anthocyanins constituted only 2.61 and 2.92% of the sum of the phenolics in FJ and PJ, respectively. Chlorogenic acid, quantitatively the main phenolic compounds in both samples, has also been demonstrated as a pancreatic lipase inhibitor with an IC_50_ value of 286.5 µM, however it has rather weak inhibitory potential compared to orlistat with an IC_50_ = 1.2 µM [31]. On the other hand, Worsztynowicz et al. demonstrated that chlorogenic acids isolated from chokeberry fruit did not inhibit the pancreatic lipase [32]. Some authors suggest that the inhibition of the pancreatic lipase by fruits is attributed to proanthocyanidins [23,28,33]. This group of polyphenols constituted 19.52% and 16.30% of the sum of the phenolics in FJ and PJ, respectively. Most likely, the anti-lipase potential of fruit extracts and juices, which are characterized by a complex phenolic composition, is the result of their mutual interactions. Other components of the samples analyzed may also influence their inhibitory activity toward pancreatic lipase. In the present study, IC_50_ values for lipase inhibition by FJ and PJ differed almost five times, despite the fact that PJ showed almost a ninety times higher concentration of phenolic compounds than FJ.

### 3.3. The Effects of V. opulus on Cellular Viability, Adipogenesis and Adipolysis

**Figure 3:**
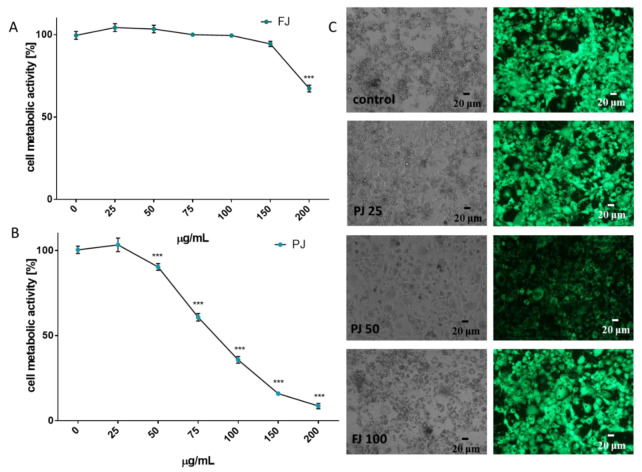
The influence of *V. opulus* FJ and PJ on 3T3L-1 cell metabolic activity determined by the PrestoBlue assay after 48 h exposure of FJ (**A**) and PJ (**B**); control cells were not exposed to any compound; values are means ± standard deviations from at least three independent experiments, *n* ≥ 12; statistical significance was calculated versus control cells (untreated), *** *p* ≤ 0.001. Morphology of 3T3L1 cells observed on the 5^th^ day of the cell differentiation process (**C)** with 25 and 50 μg/mL of PJ, and 100 μg/mL of FJ; randomly chosen fields were photographed at × 200 phase-contrast and a fluorescent microscope (cells stained with 2 µM calcein AM).

To check the influence of *V. opulus* on the metabolic activity of 3T3-L1 cells were exposed to increasing concentrations of extracts (from 10 to 200 µg/mL) for 48 h after reaching confluence. As is presented in Figure 3A, fresh juice inhibited cell viability by almost 35% at the highest concentration used. Dosages of FJ not exceeding 100 µg/mL had no cytotoxic effect on cells. Purified juice (PJ) showed higher cytotoxic potential, because its 100 µg/mL concentration decreased metabolic activity by almost 65% (Figure 3B). Within the dosages studied, the IC_50_ cytotoxicity parameter was obtained only for PJ (IC_50_ ≈ 85 µg/mL). The highest non-cytotoxic concentrations (IC_0_) chosen for studies of adipogenesis regulation were 100 μg/mL for FJ and 25 μg/mL for PJ, respectively.

The lack of cytotoxic effects of the *V. opulus* samples used at the IC_0_ concentration was also confirmed with microscopic observations of the adipocytes performed 5 days after the initiation of their differentiation (Figure 3C). Control cells appeared more rounded, had lipid droplets visible and strong cytosolic green fluorescence of calcein after staining with calcein AM ester. Simultaneously, cells incubated with PJ used in its cytotoxic concentration (50 μg/mL) had lower cytoplasmic esterase activity; thus, decreased green fluorescence of calcein was observed, and the cells were more irregular and elongated in their shape. The IC_0_ dosages obtained for FJ and PJ seem to be comparable with those previously observed for Caco-2 and MIN-6 cells; however, 3T3-L1 cells were more sensitive to the compounds studied—probably due to the longer incubation time [18,21].

**Figure 4:**
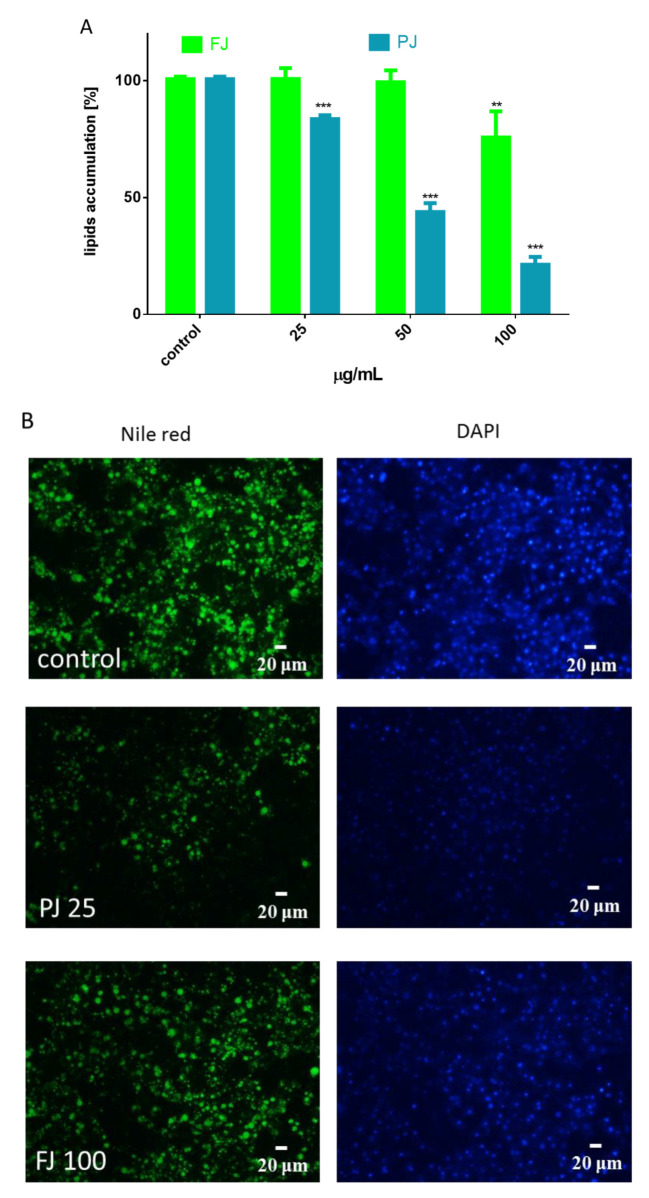
Influence of *V. opulus* FJ and PJ preparations on the accumulation of lipid droplets in 3T3-L1 cells stained with Nile red observed on the 7^th^ day of differentiation (**A**). The control cells were not exposed to any compound; the values are means ± standard deviations from at least three independent experiments, *n* ≥ 12; the statistical significance was calculated versus the control cells ** *p* ≤ 0.01, *** *p* ≤ 0.001. Cell lipid droplets with Nile red were visualized under a fluorescent microscope (200× magnification) (**B**). DAPI staining allowed for the visualization of cell nuclei with 25 μg/mL of PJ and 100 μg/mL of FJ.

The differentiation process of preadipocyte to mature adipocyte is associated with an increase of lipid droplets formation [22]. The cells staining with Nile red, a hydrophobic fluorescent dye that accumulates in neutral lipid droplets, allowed us to determine the *V. opulus* influence on cellular lipid droplets formation and accumulation (Figure 4).

**Figure 5:**
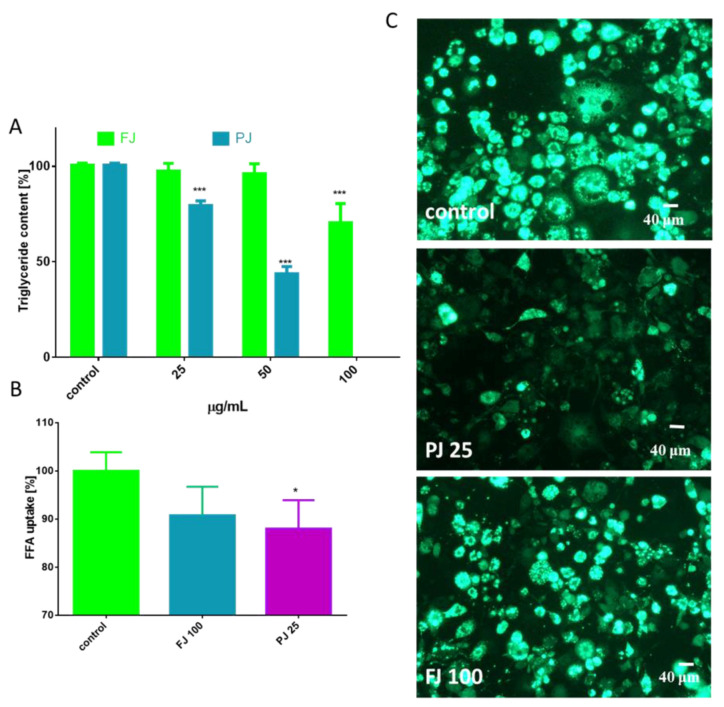
The influence *V. opulus* extracts on the triglyceride level (**A**) and fatty acid analog TF2-C12 uptake measured in 3T3L1 cells on the 7^th^ day of differentiation (**B**); control cells were not exposed to any compound; values are means ± standard deviations from at least three independent experiments, *n* ≥ 9; statistical significance was calculated versus control cells * *p* ≤ 0.05, *** *p* ≤ 0.001. Cellular uptake of FFA-C12 analogue visualized under a fluorescent microscope (400 × magnification) (**C**).

As is presented in Figure 4A, the FJ at 100 µg/mL significantly reduced the formation of lipid droplets compared to the control cells, whereas PJ was able to decrease lipid accumulation by 25% at 25 µg/mL. These data were confirmed by microscopic observation (Figure 4B). As evident by Nile red staining, PJ reduced the accumulation of lipid droplets in cytoplasm, as well as the size of droplets formed was decreased. The intracellular content of TAG was also quantified. The data show that in the presence of the preparations used at IC_0_ dosage the level of TAG was reduced by 20–25% (Figure 5A). It can be also seen that the PJ used at concentrations higher than 50 µg/mL was the most active inhibitor of lipid accumulation. However, it needs to be emphasized that observed lipid content reduction resulted from decreased cell viability (Figure 3B).

Simultaneously, the influence of FJ and PJ on cellular free fatty acid (FFA) uptake was determined. As shown in Figure 5B, the level of fluorescent free fatty acid analogue TF2-C12 incorporated in the presence of PJ was decreased by almost 10%. Cells treated with PJ lacked strong fluorescence of lipid bodies, with the fluorescent analogue present mainly in the cytoplasm (Figure 5C). Collectively, it can be concluded that the purified juice of *V. opulus* (PJ) inhibited the adipogenic differentiation of 3T3-L1 cells.

**Figure 6:**
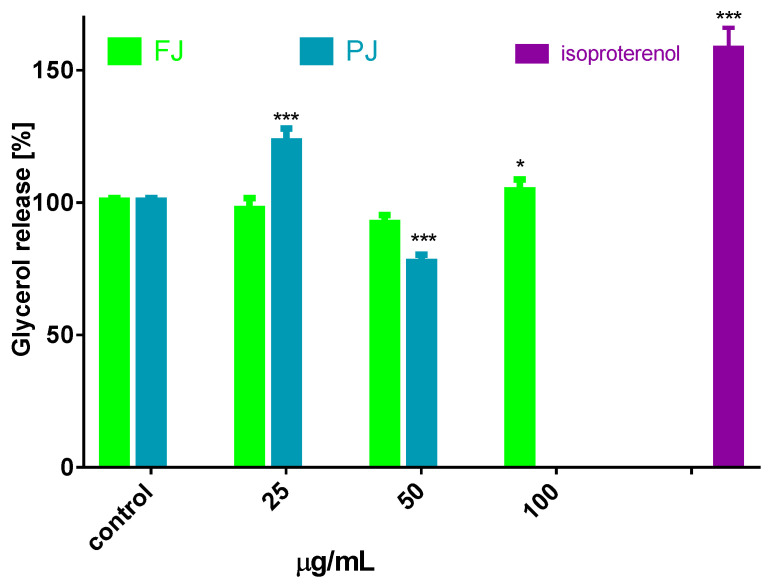
Lipolytic activity of *V. opulus* FJ and PJ preparations on differentiated 3T3L1; as a positive control 10 µM isoproterenol was used; control cells were not exposed to any compound; values are means ± standard deviations, *n* = 6; statistical significance was calculated versus control cells * *p* ≤ 0.05, *** *p* ≤ 0.001.

Besides inhibiting lipogenesis, it is possible that *V. opulus* could influence the lipolysis process, which leads to the stored TAG breakdown to fatty acids and glycerol [34]. Cell incubation with preparations increased the amount of glycerol released from adipocytes, with maximal stimulation by 20% observed for 25 µg/mL of PJ (Figure 6), while for the FJ preparation used in 100 µg/mL concentration this increase was only 7%. The present study provides evidence that *V. opulus* components may inhibit lipogenesis and stimulate adipolysis. The decrease in lipolysis observed in the presence of elevated dosages of PJ (50 µg/mL) resulted from its cytotoxic potential. Taking into account the obtained results, it can be concluded that the *V. opulus* influence is dose dependent according to the concentration of phenolic compounds. Regardless of the 90-fold higher concentration of phenolic compounds in PJ, its IC_0_ dose against 3T3-L1 cells is only 4-times lower than FJ. The most responsible for observed activity seems to be chlorogenic acid, which was identified as the main phenolic compound in probes. However, due to potential synergic activities and chemical interactions, other compounds present in juice, but lost during its purification process, such as procyanidins and proteins, are also responsible for the observed cellular effect. In this regard, the presented results are in agreement with our previous studies, where phenolic rich fraction from *V. opulus* fruit had stronger activity in Caco-2 and MIN6 cells [18,21].

### 3.4. The Effects of V. opulus on Expression of Genes Associated with Adipocyte Differentiation

**Figure 7:**
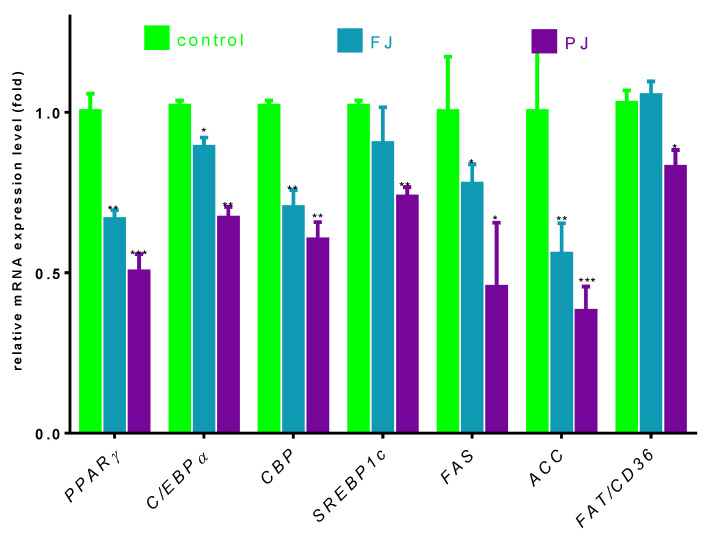
The influence of *V. opulus* FJ and PJ preparations on the gene expression in differentiated 3T3-L1 cells quantified by real-time PCR and normalized using β-actin as a reference gene. Control cells were not exposed to any compound; values are means ± standard deviations, *n* ≥ 4; statistical significance was calculated versus control cells (untreated), * *p* ≤ 0.05, ** *p* ≤ 0.01, *** *p* ≤ 0.001.

**Figure 8:**
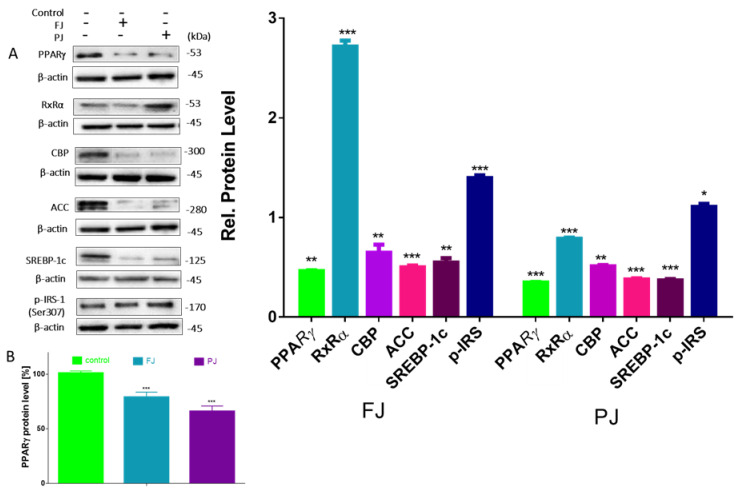
Effect of *V. opulus* FJ and PJ preparations on the relative protein level of crucial proteins involved in adipogenesis and lipogenesis determined by Western blot analysis in differentiated 3T3-L1 cells (**A**); the level of PPARγ determined with ELISA assay in nuclear fraction (**B**); control cells were not exposed to any compound; the values are means ± standard deviations, *n* ≥ 5; statistical significance was calculated between treatment and control cells (untreated), * *p* ≤ 0.05, ** *p* ≤ 0.01, *** *p* ≤ 0.001.

Differentiation of preadipocyte to adipocyte is regulated by set of transcription factors, which, upon activation, induce the expression of other adipocyte-specific genes [35]. In order to clarify the molecular effects of *V. opulus* components, the analysis of selected genes’ expressions, as well as protein levels, was performed. Given the fact that *V. opulus* diminished lipid accumulation in 3T3-L1 cells, we first checked the effects of FJ and PJ used at the IC_0_ concentration on the mRNA expression of master adipogenic regulators: peroxisome proliferator-activated receptor gamma (PPARγ), CCAAT/enhancer-binding proteins (C/EBPα) and sterol regulatory element-binding protein 1c (SREBP-1c), which have a direct impact on fat cells’ development [35]. As shown in Figure 7, both samples suppressed the expression of these factors compared to untreated cells. In regard to previous studies, the purified juice rich in phenolic compounds presented a higher impact on 3T3-L1 cells than the fresh juice. Following PJ treatment, the PPARγ mRNA level decreased to 50%, while C/EBPα, CBP and SREBP-1c mRNA levels declined by 30–40%. Among the genes studied, fresh juice had no impact on the changes of SREBP-1c mRNA expression, whereas a decrease in other genes’ levels did not exceed 30%. Furthermore, we investigated the effect of tested samples on these transcription factors’ protein level. Immunoblot analysis also revealed that the PJ reduced the amount of PPARγ protein in adipocyte to 40% as compared to the control cells (Figure 8A). It is known that the activation of PPARγ protein by specific agonists results in its translocation to the nucleus, where heterodimerizes with the retinoid x receptor alpha (RxRα) [35]. Thus, we decided to study a subcellular distribution of PPARγ receptor. The level of protein detected in the nuclear fraction of adipocytes with ELISA technique showed that, in this regard, PJ diminished PPARγ distribution inside the nucleus to 60% (Figure 8B). As was mentioned above, PPARγ transcriptional activation is initiated after its binding with the RxRα receptor in the nucleus. That step of heterodimer formation describes both proteins as ligand-activated transcription factors, which coordinately regulate the gene expression of other crucial proteins involved in fatty cells differentiation, such as adipogenesis, lipid storage, lipogenesis and thermogenesis [5,6]. The results in Figure 8 show that FJ induced nearly 3-fold increase in RxRα protein. This may be caused by the presence of retinoids and 9-cis-retinoic acid in studied preparations. These compounds are known precursors of carotenoids and were found to increase RxRα expression and activity [17,36,37,38]. The solid-phase purification of the FJ eliminated these compounds; thus, cells’ treatment with PJ showed a 20% decrease in RxRα protein. Therefore, one can conclude that the observed limitation of PPARγ-RxRα heterodimer formation in the cells incubated with *V. opulus* may also partially reduce adipogenesis.

**Figure 9:**
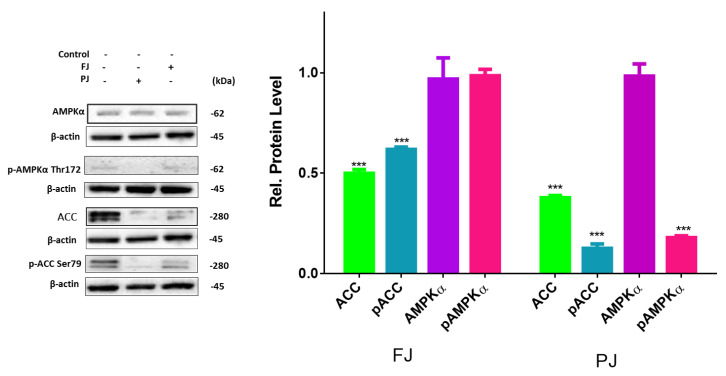
Effect of *V. opulus* FJ and PJ preparations on the relative levels of phosphorylated proteins involved in adipogenesis determined by Western blot analysis in differentiated 3T3-L1 cells. Control cells were not exposed to any compound; values are means ± standard deviations, *n* ≥ 4; statistical significance was calculated between treatment and control cells (untreated) with *** *p* ≤ 0.001.

Regardless of this, it was also demonstrated that CREB-binding protein (CBP) after its activation binds to the promoter of the C/EBP gene being transcriptional coactivator that associates with PPARγ. It has been demonstrated that IBMX and cyclic-AMP (cAMP) analogs promote adipocyte differentiation via CREB phosphorylation [39]. As before, *V. opulus* downregulated CBP on the transcriptional and translational levels (Figure 8 and Figure 9); however potential CREB phosphorylation via the cAMP-dependent pathway needs to be further elucidated.

Next, we investigated the effect of *V. opulus* FJ and PJ on the expression of genes that are up-regulated during adipocyte differentiation and controlled by the abovementioned transcription factors. Fatty acid synthase (FAS) is involved in the *de novo* synthesis of long-chain fatty acids; thus, FAS inhibition has been considered as one of the major factors decreasing the amount of intracellular fatty acids and lipogenesis [40]. While we have not directly checked the *V. opulus* influence on FAS enzymatic activity, the significant downregulation of FAS expression by both preparations was detected (Figure 7). This effect was, again, slightly stronger for the PJ than for the FJ preparation (0.55 and 0.49, respectively). In addition, 3T3-L1 cells’ incubation with PJ influenced the mRNA level of the FAT/CD36 gene. The protein encoded by this gene is involved in the transmembrane movement of fatty acids and studies performed on CD36 knockout mice showed decreased fatty acids uptake by adipocytes [41]. According to the data presented in Figure 7, the 3T3-L1 cells incubated with PJ showed that FAT/CD36 expression decreased by 20%, which matches that mechanism with previously observed diminished cellular FFA uptake. Furthermore, the reduction in SREBP-1c protein expression results in the inhibition of the expression of the enzyme catalyzing synthesis of the malonyl-CoA involved in fatty acid and triglyceride synthesis—acetyl-CoA carboxylase (ACC) [42]. The levels of the gene and ACC protein expression were decreased by almost 50% (Figure 7 and Figure 8) and these results are in agreement with the observed decrease in lipid and TAG content in mature 3T3-L1 cells. The results obtained are in line with the decrease of FFA uptake observed in Caco-2 cells [18]. However, it was also presented that *V. opulus* increased FFA uptake and lipids accumulation in insulinoma MIN6 cells, which may deleteriously effect insulin secretion [19].

Previous studies showed that, despite the activation of PPARγ, the differentiation of preadipocyte can be modulated by AMP-activated protein kinase (AMPK) involvement [43]. To clarify an influence of *V. opulus* extracts on 3T3-L1 differentiation suppression we studied whether these extracts are able to activate AMPK. One of the key factors of AMPK activation is the elevation of AMP level leading to the protein α-subunit threonine 172 phosphorylation [44]. As is shown in Figure 9, the level of total AMPK was not affected by *V. opulus* components, whereas the level of p-AMPKα was significantly decreased by the PJ preparation.

Among the AMPK substrates involved in the adipogenesis is acetyl-CoA carboxylase (ACC). The AMPK-catalyzed phosphorylation of ACC inhibits its enzymatic activity. While the mRNA and protein levels of ACC were decreased in cells treated with preparations, only the FJ elevated amount of phosphorylated ACC (Figure 9). In cells treated with the purified juice p-ACC level was notably diminished to 20% in comparison with the control cells (Figure 9), which is also in concordance with the observed decrease in p-AMPK level. In this regard it can be concluded that *V. opulus* phenolic components may inhibit activities of AMPK upstream kinases, like tumor-suppressor liver kinase B1 (LKB1), calcium-dependent calcium/calmodulin-dependent protein kinase kinase β (CaMKKβ) and transforming growth factor-β activated protein kinase-1 (TAK1), or activate mechanism independent of AMPK [45].

### 3.5. The Effects of V. opulus on Intracellular Reactive Oxygen Species Production and Selected Adipokines and Cytokines Secretion

**Figure 10:**
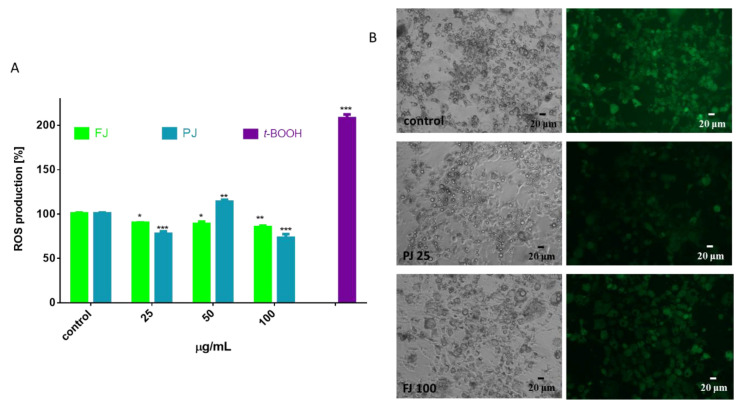
Influence of *V. opulus* FJ and PJ preparations on the intracellular reactive oxygen species generation analyzed by DCFH-DA assay in differentiated 3T3-L1 cells (**A**); control cells were not exposed to any compound; as a positive control for ROS generation, 500 µM t-BOOH was used; values are means ± standard deviations from at least three independent experiments, *n* ≥ 9; statistical significance was calculated versus control cells * *p* ≤ 0.05, ** *p* ≤ 0.01, *** *p* ≤ 0.001. Cells visualized under phase-contrast and fluorescent microscope (200× magnification) (**B**).

During the adipogenesis and adipocyte enlargement, the intracellular reactive oxygen species (ROS) generation by the mitochondria is intensified and contributes to energy metabolism [8]. Thus, we analyzed the influence of FJ and PJ on intracellular ROS formation. As is illustrated in Figure 10A, the *V. opulus* both samples used at the IC_0_ dosages decreased oxidation status in adipocytes by 10–15% as compared to the control cells. Observations with the fluorescence microscopy of cells stained with fluorogenic dichloro-dihydro-fluorescein diacetate (DCFH-DA) correspond to the presented quantitative results (Figure 10B). This cytoprotective activity may be related to the antioxidant properties of the components of the studied extracts. The main components identified in *V. opulus* fruit juice are phenolic compounds, which are involved in direct free radical quenching. Our previous studies revealed that the comparable dosages of *V. opulus* extracts were also able to decrease radicals generation and intracellular ROS level in Caco-2 and MIN6 cells [18,21]. Furthermore, *V. opulus* compounds were able to enhance activity of enzymes involved in cellular defense system, i.e., glutathione peroxidase (GPx). Nevertheless, the elevated dosage of PJ (50 µg/mL) increased intracellular oxidative stress as a result of the mitochondrial depolarization induced by sample components [21]. The observed reduction in ROS level for PJ at 100 µg/mL concentration confirms its cytotoxic ability (demonstrated previously in Figure 3), leading to a decrease in cell number and the induction of cellular death.

**Figure 11:**
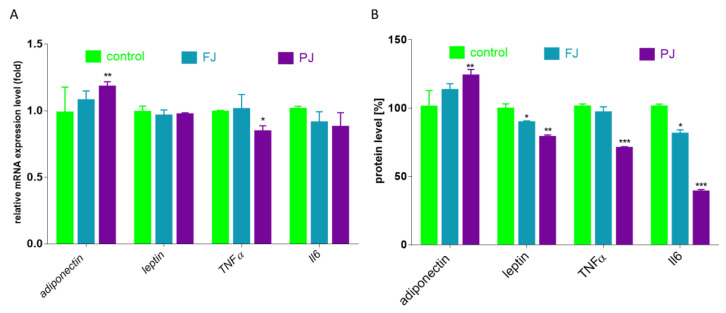
Influence of *V. opulus* FJ and PJ preparations on the mRNA expression (**A**) and protein secretion (**B**) of selected adipokines and cytokines in differentiated 3T3-L1 cells; control cells were not exposed to any compound; values are means ± standard deviations, *n* = 6; statistical significance was calculated versus control cells * *p* ≤ 0.05, ** *p* ≤ 0.01, *** *p* ≤ 0.001.

Differentiated adipocytes are able to secrete proteins that are responsible for adipose tissue remodeling, as well as inflammation generation. The most abundant adipokine is adiponectin, the levels of which are decreased in subjects with diet-related diseases, such as obesity or type 2 diabetes. Activated PPARγ, C/EBPα and SREBP1c stimulate its expression in adipocyte [7]. Adiponectin targets adiponectin receptor (AdipoR) mainly regulates energy metabolism and reveals protective insulin-sensitizing and anti-inflammatory properties. The second protein, leptin, plays role in appetite suppression and the downregulation of food intake; however, its serum concentration is increased in obesity due to observed leptin resistance [46]. As it is demonstrated in Figure 11, the incubation of differentiated 3T3-L1 cells with PJ preparation increased the expression of adiponectin gene by 15%, as well as up-regulated its extracellular secretion by 25% compared to the control cells. In the same way, there was a noticeably diminished level of lipid and TAG accumulation in 3T3-L1 cells. Despite the lack of the FJ and PJ influence on leptin mRNA expression level, there was an observed relevant decrease in leptin secretion by cells treated with these preparations (15–20%). Studies performed *in vivo* demonstrated that chlorogenic acid (dominant phenolic compound in FJ and PJ) effectively reduced blood and liver lipid accumulation, as well as decreased amounts of leptin and insulin in plasma [47]. The enlargement of adipocytes induces the release of free fatty acids, which generate oxidative stress, leading to cellular structures damage, but also stimulate the secretion of inflammatory cytokines. Among cytokines, the most related to obesity and insulin resistance are tumor necrosis factor α (TNFα) and interleukin-6 (Il-6). Chlorogenic acid has an anti-inflammatory effect, reducing the cellular release of TNFα, Il-1β and Il-6 cytokine [9,48,49,50]. Figure 11 shows that only purified juice declined the expression of TNFα mRNA by 20%. TNFα protein level was also downregulated by PJ to 70%. It is known that TNFα regulates the synthesis of pro-inflammatory cytokines, including Il-6 [51]. In concordance, both *V. opulus* preparations decreased the secretion of Il-6 protein to 40–70%. In contrast, Il-6 mRNA level was not changed. Hence, we can conclude that the FJ and PJ components could be further checked with animal models of obesity or insulin resistance as potential preventive agents against chronic inflammatory response.

To the best of our knowledge, there are no reports on *V. opulus* fruit influence on lipid metabolism regulation with PPARγ involvement. However, it is known that chlorogenic acid, the main phenolic constituent of *V. opulus*, was able to suppress mRNA level of Pparγ, Cd36 and Fabp4 in liver and white adipose tissue in mice treated with a high-fat diet [49]. The results obtained by Villalpando-Arteaga et al. are comparable and show that aqueous extract from *Hibiscus sabdariffa* containing chlorogenic acid, delphinidin-3-sambubioside and cyanidin-3-sambubioside, attenuated steatosis progression and Pparγ expression in the liver of obese mice [52]. Chlorogenic acid and rutin (quercetin-3-rutinoside) were observed as effective inhibitors of the accumulation of intracellular triglyceride content in 3T3-L1 cells [53]. They were able to down-regulate the expression of adipogenic transcription factors (PPARγ and C/EBP) and adipocyte-specific proteins (leptin), as well as up-regulate adiponectin. In comparison, other study performed with 3T3-L1 differentiated cells identified chlorogenic acid as an agonist of PPARγ2, which promoted adipocyte differentiation via the elevation of PPARγ2, CEBP and SREBP-1 mRNA and protein level [54]. Nevertheless, the other phenolic compounds identified in *V. opulus* fruit were proved to decrease the adipogenesis process [55,56,57]. Cyanidin-3-glucoside was able to elevate adiponectin gene expression in human adipocytes, whereas, in C57Bl/6J mice diminished the expression of Fas and Srebp-1 and decreased body weight and hepatic lipid content [55,57]. Reduced levels of SREBP-1c, ACC and FAS were noted in 3T3-L1 cells after treatment with quercetin derivatives and rutin [58,59]. Additionally, rutin and catechin were capable of suppressing adipocyte differentiation via PPARγ and RxRα receptor down-regulation [59,60,61]. Catechin effectively suppressed 3T3-L1 cells differentiation with the inhibition of the expression and protein levels of PPARγ and FAS; however, the observed results were stronger after the cells’ treatment with a combination of catechin and caffeine [62]. Procyanidin B2 and other derivatives present in FJ and PJ preparations were also confirmed as regulators of adipocyte triglyceride content with PPARγ involvement [63,64].

### 3.6. The Effects of V. opulus on Activity of PPARγ

**Figure 12:**
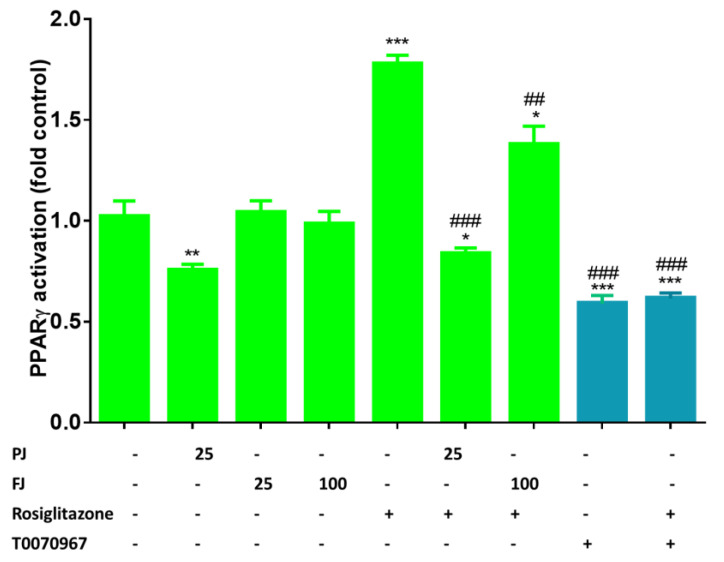
The effect of *V. opulus* FJ (25 and 100 µg/mL) and PJ (25 µg/mL) preparations on PPARγ activation in GeneBLAzer® PPAR gamma 293H DA reporter gene assay; as agonist 1 µM rosiglitazone was used, whereas as antagonist 1 µM T0070967; the control cells were not exposed to any compound; the values are means ± standard deviations, *n* = 4; the statistical significance was calculated versus control cells with * *p* ≤ 0.05, ** *p* ≤ 0.01, *** *p* ≤ 0.001; the statistical significance was calculated versus cells after rosiglitazone treatment with ^##^ *p* ≤ 0.01, ^###^ *p* ≤ 0.001.

Among different transcriptional activators of adipogenesis, PPARγ, which belongs to nuclear receptors family, is considered as a master regulator of adipocytes differentiation. It interplays with other transcription factors and binds numerous proteins involved in the regulation of transcription, such as PPARγ cofactor 1α (PGC-1α) [8,65]. PPARγ activity can be regulated by ligand binding to the ligand-binding domain, inducing protein conformational changes. Among natural precursors of ligands for PPARγ are long-chained fatty acids, as well as nitriled or oxidized lipids [66]. Thus, to detect if *V. opulus* phenolic components directly affect the activity of PPARγ protein, we used the cell-based reporter gene assay. As a PPARγ activator, we used rosiglitazone at 1 µM concentration, while compound T0070967 (1µM) was used as an antagonist. As it is shown in Figure 12, after the cells’ incubation with agonist PPARγ, activity was elevated almost twofold compared to the control cells, whereas the antagonist decreased the receptor activity by 40%. PPARγ activity was diminished by 25% after treatment with PJ, whereas FJ had no effect. In addition, the cells’ preliminary incubation with tested preparations significantly decreased receptor activity after rosiglitazone treatment by 90% and 40% for PJ and FJ, respectively. Based on this, we can suspect that *V. opulus* juice components possess antagonist potential against PPARγ receptor.

**Figure 13:**
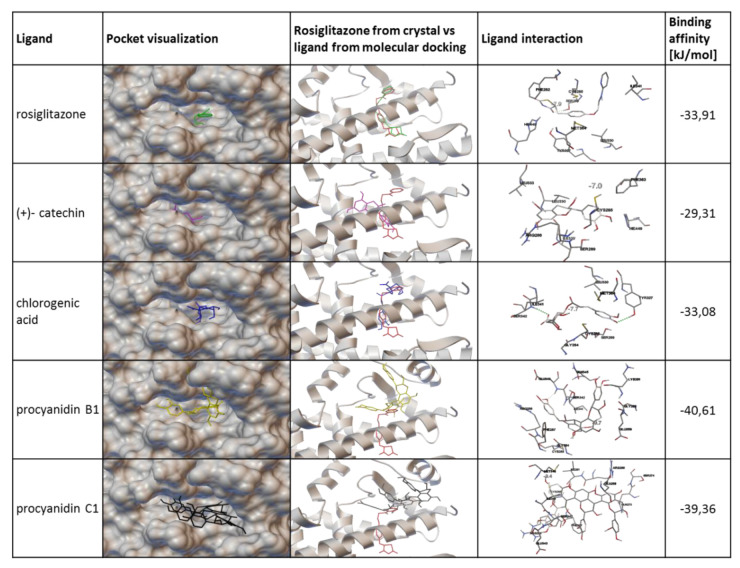
Interaction of tested phenolic compounds with PPARγ receptor verified with molecular docking. The active site of the PPARγ receptor is located behind the E3 helix, where a small cavity is formed. An agonist of PPARy, rosiglitazone, enters the receptor-active site and locates deep inside the binding pocket. Molecular models of rosiglitazone, (+)- catechin, chlorogenic acid, procyanidin B1 and procyanidin C1 are shown on the entrance of the active pocket site. Orientation of models are presented in comparison to the crystal model of rosiglitazone. Interactions of the models with corresponding binding affinities are presented in the two last columns. Molecular docking was performed with AutoDock Vina.

PPARγ protein belongs to the superfamily of nuclear receptors and possesses six domains, among which the ligand-binding domain (LBD), via interactions with ligands, is involved in the modulation of PPARγ activity. Afterwards, the ligand binding induces a change in the receptor conformation dynamic process of corepressor dissociation, and coactivator recruitment is started [14]. To explore the potential interaction between *V. opulus* juice components and PPARγ protein, molecular docking simulation was performed with the binding pocket located behind the H3 helix of PPARγ receptor. There are many PPARγ receptor agonists that locate in this binding pocket, i.e. indol-1-yl acetic acid, nonaoic acid or amorfrutin 1 [67,68,69]. As an example of the PPARγ agonist in the analyzed model, we used rosiglitazone (Figure 13), which locates deeply inside the receptor-binding pocket. The simple validation of the docking method showed slight differences between rosiglitazone in crystal structure and docked molecule in the pocket; however, this effect was acceptable for performed research with the main phenolic compounds identified in *V. opulus* juice. Molecular modeling with Autodock Vina located chlorogenic acid and (+)- catechin inside the binding pocket. Among the studied compounds, chlorogenic acid, (+)- catechin and rosiglitazone showed similar binding affinities (Figure 13). Additionally, the chlorogenic acid molecule orientation in pocket also revealed some similarity to that of rosiglitazone. Despite this, neither chlorogenic acid nor (+)- catechin did not create any possible hydrogen bond to serine 289 or tyrosine 473 of PPARγ, which were essentially made by the rosiglitazone molecule. This gives some hint to predict that these compounds have some potential to activate PPARγ, but their mechanism of action could be different than rosiglitazone; they could bind to a different part of the receptor and change its conformation in a similar way to the partial agonist. In contrast to these two phenolic ligands, procyanidins B1 and C1 cannot fit into the binding pocket because of their more complex structures and larger shape. In this case, estimated high binding affinity resulted from numerous hydrogen bonds created between procyanidins and the residues present in surface of receptor. Thus, procyanidins present in *V. opulus* juice could clog the entrance to the PPARγ binding pocket and block it from a potential agonist entering, which could resemble inverse agonist behavior. As a result, the reduction of PPARγ activity may occur despite the presence of chlorogenic acid, which was demonstrated to be a PPARγ agonist. Among the limitations of performed basic molecular docking, there is a lack of prediction of the most favorable energetic conformation for research space, as well as kinetic data describing designed configuration. Thus, further studies with kinetic modeling or isothermal titration calorimetry would give more detailed information in this regard. Still, the results obtained with molecular docking are in agreement with the biological experiments showing a reduction of signal transduction controlled by PPARγ, and, finally, a decrease in 3T3-L1 cells adipogenesis. Therefore, PPARγ transcriptional potential could be modulated on different levels: by the regulation of mRNA and protein levels, as well as conformational changes made after its binding with ligands leading to protein subcellular distribution, heterodimers’ formation, and, finally, binding with peroxisome proliferator hormone response elements present in promoters of PPAR-responsive genes. The studies performed recently identified chlorogenic acid as an agonist of the PPARγ2 receptor responsible for the enhanced differentiation of 3T3-L1 cells [54]. In the identified molecular mechanism downregulation of adipocyte differentiation-inhibitor gene, Pref1 was observed, which was accompanied by the upregulation of CEBP and SREBP-1 transcriptional factors. Microscopic observations demonstrated the elevation of PPARγ in the nucleus fraction, as well as in total cell lysate. Based on this data and our results, we can conclude that other *V. opulus* juice components, such as proanthocyanins, may be responsible for the observed PPARγ limitation.

To PPARγ endoligands belong unsaturated fatty acids or eicosanoids, whereas, among synthetic ligands, the most known are thiazolidinediones, such as rosiglitazone [5]. A recent study presented chemically pure phenolic compounds’ abilities to regulate the adipogenesis process and matched the obtained results with the molecular docking analysis of their binding affinity to PPARγ LBD [70]. As it was shown, the obtained in silico binding affinities of quercetin, apigenin, resveratrol, ellagic acid and coumaric acid to PPARγ were in agreement with these compounds’ inhibition potential of 3T3-L1 cells differentiation. However, it can still be supposed that the nuclear receptor ligand activity might be limited not only by its bioavailability, but also by its intracellular uptake.

## 4. Conclusions

**Figure 14:**
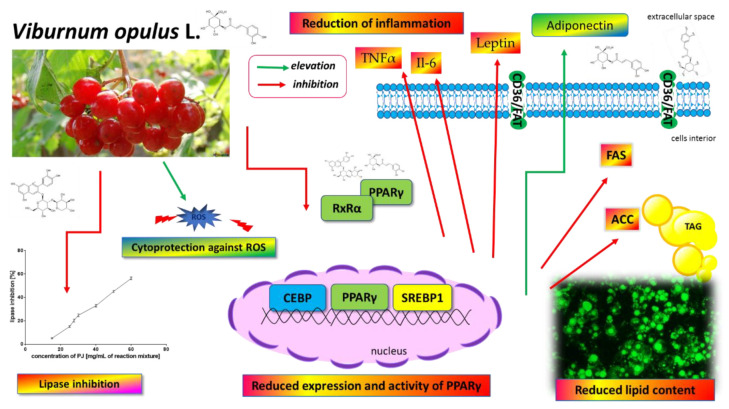
*V. opulus* juice phenolic compounds as modulators of lipids metabolism—the proposed mechanism of action. *V. opulus* downregulates the adipogenesis of 3T3-L1 cells, decreases the expression and activity of the PPARγ nuclear receptor as well as PPARγ-regulated proteins (i.e., fatty acid synthase (FAS), acetyl-CoA carboxylase (ACC)), diminishes the release of inflammatory cytokines and leptin, possesses cytoprotective activity against ROS generation and increases adiponectin secretion. *V. opulus* inhibits pancreatic-lipase and limits fatty acid release from the food matrix.

The present work provides direct evidence of the *V. opulus* juice effect on the adipogenesis of 3T3-L1 cells (Figure 14). Phenolic compounds identified in juice, mainly chlorogenic acid, procyanidins and catechins, were found to suppress adipogenesis by the downregulation of major regulators of adipogenesis, such as PPARγ, C/EBPα and SREBP-1c. The regulation of PPARγ-mediated β-lactamase expression in reporter gene assay, as well molecular docking, suggested that *V. opulus* components may work as a PPARγ antagonist. As result, the levels of enzymes involved in lipid metabolism, such as FAS or ACC, were decreased, along with adipokine TNFα, Il-6 and leptin. Additionally, *V. opulus* juice was able to inhibit pancreatic lipase, which potentially could reduce the uptake of fatty acids in the digestive tract and the storage of body fat in the adipose tissue.

Our results contribute to elucidate *V. opulus* phenolic compounds’ molecular mechanism in adipogenesis regulation. However, observed adipogenesis inhibitory outcome needs further molecular evaluation after *V. opulus* juice *in vitro* digestion and its incubation with gut microflora. These processes may greatly influence the composition of the studied phytocompounds, as well as the activity. Taking into account possible *V. opulus* cellular type-dependent *in vitro* activity, its potential usage as a diet component needs further *in vivo* studies performed with an animal obesity model to show its efficacy in the regulation of lipid metabolism at safety doses.

## Figures and Tables

**Table 1:** Retention time (Rt), wavelengths of maximum absorption (λmax), mass spectral data, and the identification and quantification of phenolic compounds in fresh juice and purified juice of *Viburnum opulus* fruit.

Peak No.	Phenolic Compound	R_t_ (min)	λ_max_ (nm)	[M − H]^−^/ [M + H]^+^ (m/z)	Fragment(s) Ions (m/z)	Content (mg/g)	
						Fresh Juice (FJ)	Purified Juice (PJ)
**1**	Neochlorogenic acid ^1^	4,50	318	353	191, 179	0.007 ± 0.001	0,215 ± 0.019
**2**	Procyanidin dimer B1 ^1^	5.12	276	577	407, 289	0.759 ± 0.003	47.596 ± 0.148
**3**	Caffeoylquinic acid derivative I ^2^	5.17	323	707	191	0.015 ± 0.000	1.289 ± 0.058
**4**	Caffeoylquinic acid derivative II ^2^	5.35	323	707	191	0.024 ± 0.002	1.051 ± 0.008
**5**	Procyanidin trimer I ^3^	5.60	278	865	577, 289, 243	0.112 ± 0.001	6.866 ± 0.342
**6**	(+)-Catechin ^1^	5.70	278	289	245, 202	0.657 ± 0.006	40.729 ± 0.596
**7**	Caffeoylquinic acid derivative III ^2^	5.74	323	707	191	0.017 ± 0.001	1.220 ± 0.020
**8**	Procyanidin trimer II ^3^	5.87	275	865	577, 289, 243	0.030 ± 0.006	2.634 ± 0.270
**9**	Chlorogenic acid ^1^	6.20	318	353	191, 707 ^a^	8.039 ± 0.145	645.492 ± 1.984
**10**	Cryptochlorogenic acid ^1^	6.50	323	353	191, 173	0.004 ± 0.000	0.484 ± 0.023
**11**	Procyanidin dimer B2 ^1^	6.61	278	577	407, 289	0.199 ± 0.002	11.540 ± 0.148
**12**	Gallocatechin gallate ^4^	6.84	280	457	169	0.031 ± 0.000	1.876 ± 0.085
**13**	Caffeoylquinic acid ^4^	7.31	313	353	191, 133, 707 ^a^	0.745 ± 0.001	44.344 ± 0.176
**14**	(−)-Epicatechin ^1^	7.40	278	289	245, 202	0.135 ± 0.002	8.002 ± 0.116
**15**	Cyanidin-3-sambubioside ^1^	7.53	515	581^+^	287	0.093 ± 0.000	7.010 ± 0.003
**16**	Cyanidin-3-glucoside ^1^	7.74	515	449^+^	287	0.139 ± 0.000	13.583 ± 0.799
**17**	Procyanidin dimer C1 ^1^	7.86	278	865	407, 289	0.033 ± 0.001	3.212 ± 0.351
**18**	B-typeprocyanidin dimer derivative I ^5^	7.96	276	739	577, 289	0.016 ± 0.000	2.071 ± 0.097
**19**	Cyanidin-3-rutinoside ^1^	8.10	516	595^+^	287	0.068 ± 0.001	5.072 ± 0.016
**20**	Procyanidin trimer III ^3^	8.32	278	865	577, 289, 243	0.032 ± 0.000	1.796 ± 0.053
**21**	Caffeoylquinic acid derivative IV ^2^	8.40	325	705	513	0.034 ± 0.000	3.306 ± 0.014
**22**	Caffeoylquinic acid derivative V ^2^	8.56	325	705	513	0.034 ± 0.000	3.268 ± 0.010
**23**	B-type procyanidin dimer derivative II ^5^	8.92	278	739	577, 289	0.035 ± 0.000	2.293 ± 0.094
**24**	Feruloylquinic acid I ^3^	9.43	325	367	193, 134	n.d.	5.722 ± 0.021
**25**	Quercetin-3-vicianoside ^6^	9.70	351	595	301, 300, 271	0.020 ± 0.000	1.266 ± 0.007
**26**	Procyanidin dimer ^3^	10.03	278	577	407, 289	0.024 ± 0.001	1.602 ± 0.258
**27**	Feruloylquinic acid II ^3^	10.13	304	367	193, 134	n.d.	0.528 ± 0.005
**28**	Quercetin-3-galactoside ^4^	10.18	365	463	300, 271	n.d.	0.149 ± 0.011
**29**	Quercetin-3-rutinoside ^1^	10.29	344	609	301, 270, 151	0.016 ± 0.000	0.921 ± 0.007
**30**	(Epi)catechin derivative I ^2^	11.13	280	451	289, 161	0.103 ± 0.001	6.998 ± 0.221
**31**	(Epi)catechin derivative II ^2^	11.27	280	451	289, 161	0.080 ± 0.001	6.006 ± 0.165
**32**	Quercetin-3-rhamnoside ^4^	11.78	344	447	301, 270, 255, 227	0.007 ± 0.000	0.491 ± 0.002

n.d.—not detected; ^a^ dimeric adduct. Results are expressed as a mean ± standard deviation (*n* = 3). The values expressed differ significantly (one-way ANOVA and Duncan’s test, *p* ≤ 0.05). 1—identification based on a comparison of retention time, UV-vis spectra (200–600 nm) and MS data for standards; 2,3,4,5—identification based on a comparison of molecular ions and typical ion fragments with published data; 2—[6], 3—[7], 4—[8], 5—[9], 6—[10].

**Table 2:** The total content of the different phenolic groups in *V. opulus* fresh juice and purified juice according to UPLC–PDA-Q/TOF-MS analysis.

Group of Phenolics	Total Content (mg/g)	
	Fresh Juice (FJ)	Purified Juice (PJ)
**Flavanols**	2.246 ± 0.014	143.210 ± 1.377
**Hydroxycinnamic acids (HCA)**	8.919 ± 0.146	706.919 ± 2.119
**>Flavonols**	0.043 ± 0.000	2.827 ± 0.026
**Anthocyanins**	0.301 ± 0.002	25.665 ± 0.812
**Total phenolics**	11.508 ± 0.154	878.632 ± 2.722

Results are expressed as a mean ± standard deviation (*n* = 3). The values differ significantly (one-way ANOVA and Duncan’s test, *p* ≤ 0.05).
